# Supplementary material for: Management practice, quality of life and associated factors in psoriasis patients attending a dermatological center in Ethiopia
Source: PLoS One. 2021 Nov 19;16(11):e0260243. doi: 10.1371/journal.pone.0260243 (PMC8604307; doi:10.1371/journal.pone.0260243)
Supplement: S1 Questionnaire — (DOCX) [file pone.0260243.s001.docx]

## Data abstraction format from patient interviews

**Part 1: Patients socio-demographic characteristics**

Card number________________Weight (in kg)_______Weight (in kg) ________

| 1 | Sex | Male 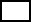 Female 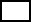 |
| --- | --- | --- |
| 2 | Marital status | Single  Married 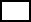  Divorced 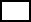  Widowed 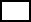 |
| 3 | Educational status | Can’t read and write 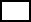  Primary education (grade 1-8) 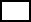  Secondary education (grade 9-12) 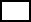  Tertiary education (diploma and above) 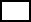 |
| 4 | Occupation | Farmer 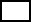  Merchant 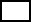  Employee 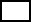  Unemployed 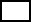  House wife 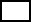  Student 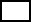  Daily laborer 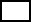  Other 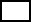 |
| 5 | Monthly family income (ETB) | Very low (≤860)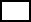  Low (861-1500) 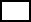  Average (1501-3000)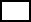  Above average (3001-5000) 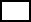  High (≥5001)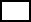 |
| 6 | Regular use of alcohol | Yes 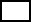 No 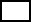 |
| 7 | Active cigarette smoker | Yes 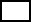 No 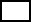 |
| 8 | Chat chewing habit | Yes 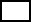 No 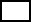 |

**Part 2: Dermatology Life Quality Index (DLQI)**

Hospital No: Date: **DLQI** **score**

Name:
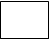


Address: Diagnosis:

**The aim of this questionnaire is to measure how much your skin problem has affected your life OVER THE LAST WEEK. Please tick one box for each question.**

| 1 | Over the last week, how **itchy, sore, painful** or **stinging** has your skin been? | Very much   A lot   A little   Not at all  | |
| --- | --- | --- | --- |
| 2 | Over the last week, how **embarrassed** or **self-conscious** have you been because of your skin? | Very much   A lot   A little   Not at all  | |
| 3 | Over the last week, how much has your skin interfered with you going **shopping** or looking after your **home** or **garden**? | Very much   A lot   A little   Not at all  | Not relevant  |
| 4 | Over the last week, how much has your skin influenced the **clothes** you wear? | Very much   A lot   A little   Not at all  | Not relevant  |
| 5 | Over the last week, how much has your skin affected any **social or leisure** activities? | Very much   A lot   A little   Not at all  | Not relevant  |
| 6 | Over the last week, how much has your skin made it difficult for you to do any **sport**? | Very much   A lot   A little   Not at all  | Not relevant  |
| 7 | Over the last week, has your skin prevented you from **working** or **studying**? | Yes   No  | Not relevant  |
|  | If "No", over the last week how much has your skin been a problem at **work or studying**? | A lot   A little   Not at all  | |
| 8 | Over the last week, how much has your skin created problems with your **partner** or any of your **close friends** or **relatives**? | Very much   A lot   A little   Not at all  | |
| 9 | Over the last week, how much has your skin caused any **sexual difficulties**? | Very much   A lot   A little   Not at all  | Not relevant  |
| 10 | Over the last week, how much of a problem has the **treatment** for your skin been, for example by making your home messy, or by taking up time? | Very much   A lot   A little   Not at all  | Not relevant  |

**Note-Please check you have answered every question. Thank you!**

©AY Finlay, GK Khan, April 1992 www.dermatology.org.uk, this must not be copied without the permission of the authors.
