# Supplementary material for: Management practice, quality of life and associated factors in psoriasis patients attending a dermatological center in Ethiopia
Source: PLoS One. 2021 Nov 19;16(11):e0260243. doi: 10.1371/journal.pone.0260243 (PMC8604307; doi:10.1371/journal.pone.0260243)
Supplement: S2 Questionnaire — (DOCX) [file pone.0260243.s002.docx]

**ክፍል 1፡ የታካሚ ማህበረሰባዊ ባህሪያቶች መረጃ**

ቀን----------------------------

የታካሚ መለያ----------------------

የካርድ ቁጥር-------------------------ክብደት (Kg) ---------------ቁመት (cm) ------------

| 1 | ጾታ | ወንድ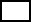ሴት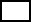 |
| --- | --- | --- |
| 2 | የጋብቻ ሁኔታ | ያላገባ/ች  ያገባ/ች 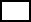  የፈታ/ች 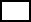  ባል የሞተባት /ሚስት የሞተችበት 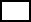 |
| 3 | የትምህርት ደረጃ | ያልተማረ/ች 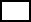  አንደኛ ደረጃ (ከ 1-8 ክፍል) 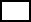  ሁለተኛ ደረጃ (ከ9-12 ክፍል) 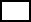  ዲፕሎማ እና ከዛ በላይ 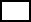 |
| 4 | የስራ ሁኔታ | ግብርና 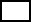  ንግድ/የግል ስራ 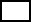  ቅጥር 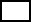  ስራ የሌለው/ላት 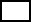  የቤት-እመቤት 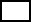  ተማሪ 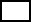  የቀንስራ 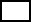  ሌሎች (ይገለጽ)---------------------------- |
| 5 | ወርሓዊ የቤተሰብ ገቢ (በብር) | በጣም ዝቅተኛ (≤860) 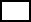  ዝቅተኛ (861-1500) 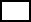  አማካይ (1501-3000) 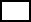  ከአማካይ በላይ (3001-5000) 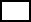  ከፍተኛ (≥5001) 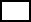 |
| 6 | ሁልግዜ/ በቋምነት ይጠጣሉ | አዎ 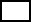 አይደለም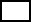 |
| 7 | ስጋራ ያጨሳሉ | አዎ 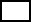 አይደለም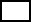 |
| 8 | ጫት ይቅማሉ | አዎ 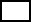 አያደለም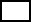 |

**ክፍል 2: Dermatology Life Quality Index: Amharic Version**

**DLQI**

የዚህ መጠይቅ አላማ የቆዳዎ ሁኔታ በአጠቃላይ ህይወት ላይ በባለፈው ሳምንት ያሳደረውን ተፅዕኖ መለካት ነው፡፡

እባክዎን ለእያንዳንዱ ጥያቄ አንድ የ
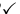
ምልክት ያድርጉ፡፡

| 1 | በባለፈው ሳምንት ሰውነትዎ ምን ያህል ማሳከክ፣ቁስለት፣ህመም እና የማቃጠል ስሜት ነበረው? | እጅግ በጣም  በጣም  በመጠኑ  በፍጹም | 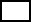  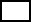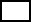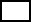 |  |
| --- | --- | --- | --- | --- |
| 2 | በባለፈው ሳምንት የቆዳዎ በሽታ ምን ያሕል አሳስብዎት እና አስጨንቆት ነበር? | እጅግ በጣም  በጣም  በመጠኑ  በፍጹም | 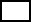  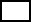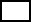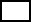 |  |
| 3 | በባለፈው ሳምንት የቆዳዎ በሽታ ገበያ ሲሄዱ አንዳንድ ስራዎችን በቤት ውስጥ ሲያከናውኑ ምን ያህል ችግር ፈጥሮዎብዎታል? | እጅግ በጣም  በጣም  በመጠኑ  በፍጹም | 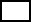  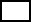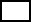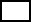 | ግኑኝነት የለውም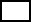 |
| 4 | በባለፈው ሳምንት የቆዳዎ በሽታ የሚለብሱትን ልብስ ምርጫ ምን ያህል ተፅዕኖ ፈጥሮቦታል? | እጅግ በጣም  በጣም  በመጠኑ  በፍጹም | 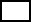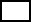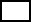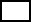 | ግኑኝነትየለውም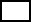 |
| 5 | በባለፈው ሳምንት የቆዳዎ በሽታ የሚያደርጉትን ማህበራዊ እንቅስቃሴ እና መዝናናት ሁኔታ ላይ ምን ያህል ተጽዕኖ ፈጥሮቦዎታል? | እጅግ በጣም  በጣም  በመጠኑ  በፍጹም | 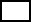  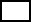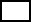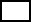 | ግኑኝነትየለውም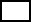 |
| 6 | በባለፈው ሳምንት የቆዳዎ በሽታ የስፖርት እንቅስቃሴ እንዳያደርጉ ምን ያሕል ተጽዕኖ ፈጥሮብዎታል? | እጅግ በጣም  በጣም  በመጠኑ  በፍጹም | 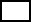  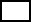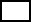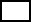 | ግኑኝነት የለውም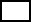 |
| 7 | በባለፈው ሳምንት የቆዳዎ በሽታ ከትምህርት ወይም ከስራ ምን ያህል አግዶዎታል? | አዎ  አይደለም | 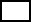  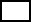 | ግኑኝነት የለውም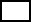 |
|  | አይደለም ከተባለ ምን ያህል በስራዎ እና ትምህርትዎ ላይ ችግር ፈጥሮብዎታል? | በጣም  በመጠኑ  በፍጹም | 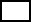  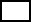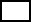 |  |
| 8 | በባለፈው ሳምንት የቆዳዎ በሽታ በርሶ የቅርብ ዘመድ ወይም ጓደኞች ጋር ባለዎት ግንኙነት ላይ የፈጠረብዎት ችግር ምን ይመስላል? | እጅግ በጣም  በጣም  በመጠኑ  በፍጹም | 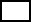  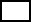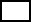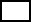 | ግኑኝነት የለውም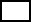 |
| 9 | በባለፈው ሳምንት የቆዳ በሽታዎ በተቃራኒ ፆታ ግንኙነት ላይ  የፈጠረብዎት ችግር ምን ይመስላል? | እጅግ በጣም  በጣም  በመጠኑ  በፍጹም | 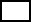  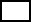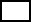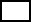 | ግኑኝነት የለውም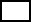 |
| 10 | በባለፈው ሳምንት የህክምናዎ ሁኔታ ያደረሰብዎት ተጽዕኖ ምን ይመስላል ለምሳሌ ቤትዎን በማመሳቀል ወይም ብዙ ጊዜ በመውሰድ ሊሆን ይችላል? | እጅግ በጣም  በጣም  በመጠኑ  በፍጹም | 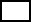  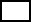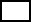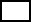 | ግኑኝነት የለውም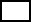 |

***© Dermatology Life Quality Index. A Y Finlay, G K Khan, April 1992***

***እባክዎንሁሉንምጥያቄዎችመመለስዎንያረጋግጡእናመሰግናለን::***
