# Supplementary material for: Management practice, quality of life and associated factors in psoriasis patients attending a dermatological center in Ethiopia
Source: PLoS One. 2021 Nov 19;16(11):e0260243. doi: 10.1371/journal.pone.0260243 (PMC8604307; doi:10.1371/journal.pone.0260243)
Supplement: S1 File — (DOCX) [file pone.0260243.s003.docx]

## Data Abstraction Format from Patient Medical Records

Date_____________

Chart code________

Patient name (initials)________Card no.__________

| 1 | Age at initial diagnosis (in years) | __________ |
| --- | --- | --- |
| 2 | Duration of the disease (months) | _________ |
| 3 | Diagnosis (types of psoriasis) | Plaque 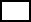  Palmoplantar 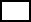  Sebopsoriasis 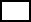  Scalp psoriasis 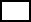  Nail psoriasis 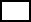  Inverse psoriasis 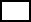  Pustular psoriasis 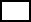  Other (specify) ________________________ |
| 4 | Comorbid disease | Present 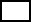  Absent 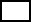 |
| 5 | If ‘**present’** to question number 5, what is the specific comorbid disease? | Metabolic syndrome 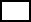  Dyslipidemia 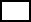  Obesity 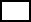  Hypertension 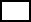  Diabetes mellitus 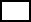  Mental stress 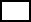  Psoriatic arthritis 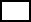  Rheumatoid arthritis 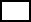  HIV 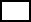  Other 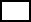 |
| 6 | Treatment type | Topical 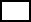  Systemic 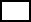 |
| 7 | Specific drugs the patient is currently taking | 1. **Topical corticosteroids**   Bethamethasone dipropoinate 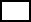  Clobetasol dipropoinate 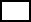  Clocortolone pivalate 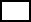  Mometasone furoate 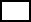  Others (specify) ______________________   1. **Keratolytic agents**   Salicylic acid 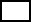  Coal tar 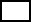  Others (specify) ______________________   1. **Systemic therapy**   Methotrexate 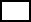  Others (specify) ________________________   1. **Medicated shampoo**   2% ketoconazole shampoo 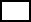  Others (specify) ________________________ |

| S. N | Laboratory investigations | Normal range (Unit) | **Date of test** | | | | |
| --- | --- | --- | --- | --- | --- | --- | --- |
|  |  |  |  |  |  |  |  |
| ***Complete blood count*** | | | | | | | |
| 1 | WBC |  |  |  |  |  |  |
| 2 | Lymphocytes |  |  |  |  |  |  |
| 3 | Neutrophils |  |  |  |  |  |  |
| 4 | PLT count |  |  |  |  |  |  |
| 5 | RBC |  |  |  |  |  |  |
| 6 | Hgb |  |  |  |  |  |  |
| 7 | Hct |  |  |  |  |  |  |
| 8 | MCV |  |  |  |  |  |  |
| 9 | MCH |  |  |  |  |  |  |
| 10 | MCHC |  |  |  |  |  |  |
| **Renal function test** | | | | | | | |
| 1 | Urea |  |  |  |  |  |  |
| 2 | Cr |  |  |  |  |  |  |
| **Liver function test** | | | | | | | |
| 1 | AST |  |  |  |  |  |  |
| 2 | ALT |  |  |  |  |  |  |
| 3 | ALP |  |  |  |  |  |  |
