# Supplementary material for: Management practice, quality of life and associated factors in psoriasis patients attending a dermatological center in Ethiopia
Source: PLoS One. 2021 Nov 19;16(11):e0260243. doi: 10.1371/journal.pone.0260243 (PMC8604307; doi:10.1371/journal.pone.0260243)

PLOS ONE

Management practice, and quality of life and its associated factors among ambulatory psoriatic patients attending Dermatological Center in Addis Ababa, Ethiopia

--Manuscript Draft--

**Manuscript Number:**	PONE-D-20-33787
**Article Type:**	Research Article
**Full Title:**	Management practice, and quality of life and its associated factors among ambulatory psoriatic patients attending Dermatological Center in Addis Ababa, Ethiopia
**Short Title:**	Management practice, and quality of life and its associated factors inpsoriatic patients
**Corresponding Author:**	Tamrat Assefa Tadesse<br>School of Pharmacy College of Health Sciences Addis Ababa University<br>Addis Ababa, ETHIOPIA
**Keywords:**	psoriasis, Dermatology Life Quality Index, quality of life, Ethiopia
**Abstract:**	Background: Psoriasis is a chronic inflammatory disease characterized by keratinocyte hyperproliferation and aberrant differentiation with great negative impact on patients' quality of life (QoL). This study aimed at assessing management practice, and quality of life (QoL) and its associated factors among ambulatory psoriatic patients visiting All Africa Leprosy, Tuberculosis and Rehabilitation Training (ALERT) Center in Addis Ababa, Ethiopia.<br>Materials and methods: A cross sectional study was conducted among 207 patients with psoriasis attending the dermatology clinic of ALERT Center in Addis Ababa, Ethiopia. Data were collected using structured questionnaire and patients' chart review. Dermatology Life Quality Index (DLQI) was used to measure patients' QoL. Data was analyzed entered and analyzed using Statistical Package for Social Sciences (SPSS®) version 25. Descriptive statistics like frequency, percent, mean and standard deviation were used to summarize patients' characteristics while binary logistic regression was utilized to identify predictors of quality of life.<br>Results : Among 207 study participants, 122(58.9%) were females. The mean age of the study population was 37.92(SD=14.86) years (ranging from 16 to 68 years). The mean age at which diagnosis of psoriasis made was 32.0(SD=13.7) years ranging from 10 to 62 years. The duration of the disease in 112(54.1%) patients were more than or equal to 5 years. Among study participants, the majority of them, 145(70.0%) had plaque psoriasis followed by sebopsoriasis, 24(11.6%). The majority of plaque psoriasis (80%) cases were managed by topical corticosteroids with or without salicylic acid or coal tar and only 21(14.5%) treated by methotrexate alone. Other forms of psoriasis were treated by only topical corticosteroids with or without salicylic acid. The mean DLQI was 6.25 corresponding to a moderate effect. Symptoms and feelings were the most affected domains of QoL. Factors associated with poor QoL were female gender [AOR=0.17 (95%CI: 0.06, 0.48)], low, above average and high family income ([AOR=0.12(95% CI: 0.02, 0.56)], [AOR=0.06(95% CI:0.01, 0.32)], and [AOR=0.03 (95% CI: 0.01, 0.22)]) respectively and primary education level [AOR=0.14 (95% CI: 0.03, 0.64)] while being on systemic therapy [AOR=4.26 (CI: 1.18, 15.35)] was predictor of better QoL.<br>Conclusion : Topical corticosteroids were the mainstay of psoriasis treatment in our study. The mean DLQI was 6.25, corresponding to a moderate effect on QoL. Study participants with female gender, primary education level, and low, above average, and high monthly family income were considered to have worse QoL whereas receiving systemic therapy significantly associated with better QoL. Hence, future interventions targeting improving QoL and widening treatment options for psoriasis deemed to be necessary.
**Order of Authors:**	Seifu Megersa Kumsa
	Tamrat Assefa Tadesse
	Minyahil Alebachew Woldu
**Additional Information:**	
**Question**	**Response**

Powered by Editorial Manager® and ProduXion Manager® from Aries Systems Corporation

**Financial Disclosure**<br><br>Enter a financial disclosure statement that describes the sources of funding for the work included in this submission. Review the submission guidelines for detailed requirements. View published research articles from *PLOS ONE* for specific examples.<br><br>This statement is required for submission and **will appear in the published article** if the submission is accepted. Please make sure it is accurate.<br><br>**Unfunded studies**<br>Enter: *The author(s) received no specific funding for this work.*<br><br>**Funded studies**<br>Enter a statement with the following details:<br>• Initials of the authors who received each award<br>• Grant numbers awarded to each author<br>• The full name of each funder<br>• URL of each funder website<br>• Did the sponsors or funders play any role in the study design, data collection and analysis, decision to publish, or preparation of the manuscript?<br>• **NO** - Include this sentence at the end of your statement: *The funders had no role in study design, data collection and analysis, decision to publish, or preparation of the manuscript.*<br>• **YES** - Specify the role(s) played.<br><br>* typeset	The author(s) received no specific funding for this work.
**Competing Interests**<br><br>Use the instructions below to enter a competing interest statement for this submission. On behalf of all authors, disclose any competing interests that could be perceived to bias this work—acknowledging all financial support and any other relevant financial or non-financial competing interests.<br><br>This statement **will appear in the published article** if the submission is accepted. Please make sure it is	The authors have declared that no competing interests exist.

accurate. View published research articles from *PLOS ONE* for specific examples.<br><br>**NO authors have competing interests**<br><br>Enter: *The authors have declared that no competing interests exist.*<br><br>**Authors with competing interests**<br><br>Enter competing interest details beginning with this statement:<br><br>*I have read the journal's policy and the authors of this manuscript have the following competing interests: [insert competing interests here]*<br><br>* typeset	
**Ethics Statement**<br><br>Enter an ethics statement for this submission. This statement is required if the study involved:<br><br>• Human participants<br>• Human specimens or tissue<br>• Vertebrate animals or cephalopods<br>• Vertebrate embryos or tissues<br>• Field research<br><br>Write "N/A" if the submission does not require an ethics statement.<br><br>General guidance is provided below. Consult the submission guidelines for detailed instructions. **Make sure that all information entered here is included in the Methods section of the manuscript.**	Ethical approval was received from the Ethical Review Board of the School of Pharmacy, College of Health Sciences, Addis Ababa University (Ref No: ERB/SOP/103/06/2019), and then permission was also obtained from ALERT Hospital, Department of Dermatology. In addition, informed written consent was obtained from the study participants and confidentiality was maintained by omitting patient identifiers and giving code number.

**Format for specific study types**<br><br>**Human Subject Research (involving human participants and/or tissue)**<br>• Give the name of the institutional review board or ethics committee that approved the study<br>• Include the approval number and/or a statement indicating approval of this research<br>• Indicate the form of consent obtained (written/oral) or the reason that consent was not obtained (e.g. the data were analyzed anonymously)<br><br>**Animal Research (involving vertebrate animals, embryos or tissues)**<br>• Provide the name of the Institutional Animal Care and Use Committee (IACUC) or other relevant ethics board that reviewed the study protocol, and indicate whether they approved this research or granted a formal waiver of ethical approval<br>• Include an approval number if one was obtained<br>• If the study involved *non-human primates*, add *additional details* about animal welfare and steps taken to ameliorate suffering<br>• If anesthesia, euthanasia, or any kind of animal sacrifice is part of the study, include briefly which substances and/or methods were applied<br><br>**Field Research**<br><br>Include the following details if this study involves the collection of plant, animal, or other materials from a natural setting:<br>• Field permit number<br>• Name of the institution or relevant body that granted permission	
**Data Availability**<br><br>Authors are required to make all data underlying the findings described fully available, without restriction, and from the time of publication. PLOS allows rare exceptions to address legal and ethical concerns. See the PLOS Data Policy and FAQ for detailed information.	Yes - all data are fully available without restriction

A Data Availability Statement describing where the data can be found is required at submission. Your answers to this question constitute the Data Availability Statement and **will be published in the article**, if accepted.<br><br>**Important:** Stating 'data available on request from the author' is not sufficient. If your data are only available upon request, select 'No' for the first question and explain your exceptional situation in the text box.<br><br>Do the authors confirm that all data underlying the findings described in their manuscript are fully available without restriction?	
**Describe where the data may be found in full sentences. If you are copying our sample text, replace any instances of XXX with the appropriate details.**<br><br>• If the data are **held or will be held in a public repository**, include URLs, accession numbers or DOIs. If this information will only be available after acceptance, indicate this by ticking the box below. For example: *All XXX files are available from the XXX database (accession number(s) XXX, XXX.*).<br>• If the data are all contained **within the manuscript and/or Supporting Information files**, enter the following: *All relevant data are within the manuscript and its Supporting Information files.*<br>• If neither of these applies but you are able to provide **details of access elsewhere**, with or without limitations, please do so. For example:<br><br>*Data cannot be shared publicly because of [XXX]. Data are available from the XXX Institutional Data Access / Ethics Committee (contact via XXX) for researchers who meet the criteria for access to confidential data.*<br><br>*The data underlying the results presented in the study are available from (include the name of the third party*	All relevant data are within the manuscript.

and contact information or URL).<br>• This text is appropriate if the data are owned by a third party and authors do not have permission to share the data.<br><br>* typeset	
Additional data availability information:	

Manuscript

Click here to access/download;Manuscript;Manuscript-Psoriasis.docx

Management practice, and quality of life and its associated factors among ambulatory psoriatic patients attending Dermatological Center in Addis Ababa, Ethiopia

Seifu Megersa Kumsa, Tamrat Assefa Tadesse, Minyahil Alebachew Woldu

Department of Pharmacology and Clinical Pharmacy, School of Pharmacy, College of Health Sciences, Addis Ababa University, Addis Ababa, Ethiopia

*Corresponding Author:

Email: tamraat.assefa@aau.edu.et

These authors contributed equally to this work.

These authors also contributed equally to this work.

Abstract

Background: Psoriasis is a chronic inflammatory disease characterized by keratinocyte hyperproliferation and aberrant differentiation with great negative impact on patients' quality of life (QoL). This study aimed at assessing management practice, and quality of life (QoL) and its associated factors among ambulatory psoriatic patients visiting All Africa Leprosy, Tuberculosis and Rehabilitation Training (ALERT) Center in Addis Ababa, Ethiopia.

Materials and methods: A cross sectional study was conducted among 207 patients with psoriasis attending the dermatology clinic of ALERT Center in Addis Ababa, Ethiopia. Data were collected using structured questionnaire and patients' chart review. Dermatology Life Quality Index (DLQI) was used to measure patients' QoL. Data was analyzed entered and analyzed using Statistical Package for Social Sciences (SPSS®) version 25. Descriptive statistics like frequency, percent, mean and standard deviation were used to summarize patients' characteristics while binary logistic regression was utilized to identify predictors of quality of life.

Results: Among 207 study participants, 122(58.9%) were females. The mean age of the study population was 37.92(SD=14.86) years (ranging from 16 to 68 years). The mean age at which diagnosis of psoriasis made was 32.0(SD=13.7) years ranging from 10 to 62 years. The duration of the disease in 112(54.1%) patients were more than or equal to 5 years. Among study participants, the majority of them, 145(70.0%) had plaque psoriasis followed by sebopsoriasis, 24(11.6%). The majority of plaque psoriasis (80%) cases were managed by topical corticosteroids with or without salicylic acid or coal tar and only 21(14.5%) treated by methotrexate alone. Other forms of psoriasis were treated by only topical corticosteroids with or without salicylic acid. The mean DLQI was 6.25 corresponding to a moderate effect. Symptoms and feelings were the most affected domains of QoL. Factors associated with poor QoL were female gender [AOR=0.17 (95%CI: 0.06, 0.48)], low, above average and high family income ([AOR=0.12(95% CI: 0.02, 0.56)], [AOR=0.06(95% CI:0.01, 0.32)], and [AOR=0.03 (95% CI: 0.01, 0.22)]) respectively and primary education level [AOR=0.14 (95% CI: 0.03, 0.64)] while being on systemic therapy [AOR=4.26 (CI: 1.18, 15.35)] was predictor of better QoL.

Conclusion: Topical corticosteroids were the mainstay of psoriasis treatment in our study. The mean DLQI was 6.25, corresponding to a moderate effect on QoL. Study participants with

female gender, primary education level, and low, above average, and high monthly family income were considered to have worse QoL whereas receiving systemic therapy significantly associated with better QoL. Hence, future interventions targeting improving QoL and widening treatment options for psoriasis deemed to be necessary.
Keywords: psoriasis, Dermatology Life Quality Index, psoriasis, quality of life, Ethiopia

Introduction

Psoriasis is a chronic inflammatory disease affecting skin and joints that is typically characterized by erythematous papules and plaques [1]. It is also associated with an array of significant medical and psychiatric comorbidities, including psoriatic arthritis, cardiovascular disease, diabetes, malignancy, depression, and anxiety that require timely therapy to improve long-term outcomes [2,3]. It is subject to flares and remissions that come in many different variations and degrees of severity which may be symptomatic throughout life and may be progressive with age or wax and wane in its severity [4].

Based on morphologic characteristics, several subtypes of psoriasis have been identified which is necessary to guide appropriate management. However, clinical findings often fit into more than one category. The most common subtypes are plaque, guttate, erythrodermic, pustular, and inverse psoriasis [5,6]. Psoriasis is a worldwide disease, despite its prevalence varies among different ethnicities and geographic locations, affecting about 2%–3% of the world’s population [7]. Higher altitudes are associated with the largest prevalence. Northern Europe appears to be one of the highest whereas East Asia tends to have the lowest rates. Similarly, global reports on psoriasis showed that prevalence varies from 0.09% to 11.4% [7,8].

The treatment of psoriasis comprises topical agents, phototherapy, systemic immunosuppressants, and, more recently, biologics [9]. Topical agents are widely used for localized and milder forms of these diseases and to control flares of skin disease in patients with widespread or more severe one while systemic therapies are reserved for more severe disease as long term use has significant side effects, despite a number of immunobiologics introduced recently has high efficacy with reduced side-effect potential [10].

Psoriasis has a negative impact on the psychological status, social interaction, and overall quality of life (QoL) of the patients [11,1]. Various studies showed that psoriasis affects day-to-day activities, occupational, and sexual functioning, often independent of the extent, and severity of skin lesions [12-15]. It was also demonstrated that it can cause anxiety, depression, anger, and embarrassment, which then leads to social isolation and absenteeism at work and school. Social and sporting activities can become difficult since they worry about what other people think of their appearance (14). It has also great economic and financial consequences [15].

There are unmet needs regarding the management of psoriasis in Africa [16]. Also, there is no generally accepted consensus definition to say the treatment is successful or not, so that patient care should include the provision of effective, convenient, and safe drugs, patient-reported outcomes like treatment preference, satisfaction, and QoL [17]. In Ethiopia, little is known about the level of quality of life and its determinants in psoriatic patients. Therefore, this study seeks to evaluate management practice, quality of life, and its associated factors among ambulatory psoriatic patients attending ALERT Hospital.

Materials and methods

Study setting

The study was conducted in the dermatology clinic of All Africa Leprosy, Tuberculosis and Rehabilitation Training Center (ALERT), located in Addis Ababa, Ethiopia which was established by the Sudan Interior Mission in 1934. ALERT Center is one of the leading specialized hospitals in dermatology, ophthalmology, and plastic and reconstructive surgery in the country.

Study design and period

Hospital-based cross-sectional study design that involved interviewing patients and reviewing their charts retrospectively was used in this study. Patients' interview was conducted from 1st July to December 31, 2019, while data extraction period was from December 31, 2018 to December 31, 2019 for the same patients.

Eligibility criteria

All patients with psoriasis and visiting dermatologic clinic during the study period and patients with age ≥16 years, who were on active follow-up and receiving treatment for at least 6 months were included in our study. Whereas being physically ill, having ≥2 questions not answered in DLQI questionnaires, and unable to speak Amharic or didn't have a caregiver to facilitate communication in case of difficulty, patients having incomplete medical records were excluded from this study.

Sample size and sampling technique

The sample size was computed based on a single population proportion formula. Since there was no previous study conducted on the QOL among psoriasis in Ethiopia, the proportion was taken as 50 % and accordingly sample size was calculated to be 384. However, during the study period (1st July to December 31, 2019), 504 patients (<10,000) visited the clinic i.e. about 21 patients; the finite population correction formula is used to calculate the actual sample size which was 218. Taking a 10% contingency (non-response rate), the final sample became 240 in our study. A systematic random sampling method was used to recruit samples for the study on each day of the data collection process. Sampling interval (kth) was determined by dividing the total number of psoriatic patients that came within the study period (six months) by estimated sample size (kth

=504/240=2), thus, the first patient was selected randomly then every other patient was interviewed following physicians visit until the required sample was reached. In this study, 207 study participants were included in the final analysis after excluding patients with incomplete data.

Study variables

QOL and management practice were considered as dependent variables while age, sex, educational status, marital status, occupation, monthly income, social drug habits, comorbidity, type of psoriasis, treatment modality, duration of psoriasis treatment, type and number of drugs were independent variables affecting QOL in this study.

Data collection tool

Data were collected using a structured data abstraction tool through interviewing patients and reviewing their medical records. The first part of the tool comprised socio-demographic character (age, sex, educational status, monthly income, occupation, alcohol intake, chat chewing, and smoking status). The second part was the DLQI questionnaire which was used to measure QoL. The final section comprised a tool to collect relevant clinical data like types of psoriasis, age at initial diagnosis, duration of disease, current medications, and comorbidities. The first part was translated into Amharic language and the Amharic version was administered to study participants.

The DLQI questionnaire was the first dermatology-specific quality of life questionnaire. It consists of questions concerning patients' perception of the impact of skin diseases on different aspects of their health-related quality of life over the last week. It was widely used in clinical studies of more than 80 countries and translated into more than 110 languages including Amharic

[18]. DLQI is a 10-item questionnaire were questions 1 and 2 assess symptoms and feelings; 3 and 4, daily activities; 5 and 6, leisure; 7, work or school; 8 and 9, personal relationships; and 10, treatment. The DLQI was rated on a 4-point scale with corresponding values of 3 = very much, 2 = a lot, 1 = a little and 0 = not at all; 0 = not relevant for each item. The highest sum is 30, and the lowest 0, and interpretation is: 0 to1 score = no effect; 2 to 5 = small effect; 6 to 10 = moderate effect; 11 to 20 = large effect and 21 to 30 = extremely serious effect on patient's life.

Data collectors and quality assurance

Data were collected by two nurses working in the outpatient dermatologic clinic under the supervision of the principal investigator. The principal investigator gave one-day training for data collectors on the study's purpose, how to conduct a patient interview, and collect data from the patients' charts. A pretest was done on twenty patients before actual data collection to check uniformity and understandability of the data collection tool and necessary modifications were made to the data collection tool. The pre-tested patients were excluded from the analysis. The principal investigator closely supervised the data collection process and was giving feedback and correction daily to maintain the quality of data.

Data analysis

After cleaning incomplete data, collected data was entered and analyzed using Statistical Package for Social Sciences (SPSS) version 25. Descriptive statistics were used for analyses of socio-demographic variables and relevant clinical data. Categorical variables were described by frequencies and percentages, and continuous variables were expressed by means and standard deviations. Binary logistic regression analysis was used to identify the determinants of QoL.

Variables with P<0.25 in univariate analysis were further analyzed by multiple logistic regressions to avoid confounders. A p-value < 0.05 was used to confirm an association.

Ethical considerations

Ethical approval was received from the Ethical Review Board of the School of Pharmacy, College of Health Sciences, Addis Ababa University (Ref No: ERB/SOP/103/06/2019), and then permission was also obtained from ALERT Hospital, Department of Dermatology. In addition, informed written consent was obtained from the study participants and confidentiality was maintained by omitting patient identifiers and giving code number.

Results

Demographic characteristics of patients

A total number of 207 participants were included for the final analysis. One hundred twenty-two (58.9%) were females. The mean age of the study population was 37.92 (SD= 14.86) years (ranging from 16 to 68 years) (Table 1).

Table 1: Socio-demographic characteristics of psoriasis patients attending Dermatology Clinic of ALERT Center, Addis Ababa, Ethiopia (N=207).

Socio-demographic	Category	Number (%)
Sex	Male	85 (41.1)
	Female	122 (58.9)
Age (in years)	16-35	106 (51.2)
	36-60	75 (36.2)
	>60	26 (12.6)
Marital status	Single	61 (29.5)
	Married	124 (59.9)

	Divorced	6 (2.9)
	Widowed	16 (7.7)
Educational status	Unable to read and write	39 (18.8)
	Primary school (Grade 1-8)	46 (22.2)
	Secondary school (Grade 9-12)	44 (21.3)
	Higher education (Diploma and above)	78 (37.7)
Occupation	Farmer	24 (11.6)
	Merchant/self-employed	46 (22.2)
	Employee	53 (25.6)
	Unemployed	14 (6.8)
	Housewife	22 (10.6)
	Student	29 (14.0)
	Daily laborer	10 (4.8)
	Others*	9 (4.3)
Monthly family income (ETB)**	Very low (≤860)	32 (15.5)
	Low (861-1500)	42 (20.3)
	Average (1501-3000)	24 (11.6)
	Above average (3001-5000)	32 (15.5)
	High (≥5001)	77 (37.2)
Social drug habits	Active cigarette smoker	3 (1.4)
	Regular alcohol user	7 (3.4)
	Regular chat chewer	11 (5.3)

ETB- Ethiopian birr; *retired, broker, guard, ** based on Ethiopian civil service monthly salary scale for civil servants

In this study, 124 (59.9%) were married participants and 78 (37.7%) participants were educated up to diploma and above. Of the total patients, 53(25.6%) were employees followed by merchant/self-employed 46(22.2%), and 77(37.2%) patients had a high level of monthly family income.

Clinical characteristics of patients

As shown in Table 2, the mean age at which the diagnosis of psoriasis made was 32.0 [(standard deviation (SD)=13.7] years. The duration of the disease in 112(54.1%) patients were more than or equal to 5 years.

Table 2: Clinical characteristics of psoriasis patient attending dermatology clinic of ALERT Center, Addis Ababa, Ethiopia (N=207).

**Factors**	**Category**	**N (%)**	**Mean± SD**	**Range**
BMI (Kg/m^2)	≤18.5	15 (7.2)		
	18.5-24.9	158 (76.3)		
	25.0-29.9	17 (8.2)		
Age at initial diagnosis (in years)	Early onset (<40)	144 (69.6)	32.0±13.7	10.0-62.0
	Late-onset (≥40)	63(30.4)		
Duration of the disease (in years)	<5	95 (45.9)	5.6±3.6	0.8-18.7
	≥ 5	112 (54.1)		
Types of psoriasis	Plaque psoriasis	145(70.0)		
	Sebopsoriasis	24 (11.6)		
	Scalp psoriasis	22 (10.6)		
	Palmoplantar psoriasis	13 (6.3)		
	Pustular psoriasis	2 (1.0)		
	Inverse and nail psoriasis	1 (0.5)		
Comorbid diseases	Yes	6 (2.9)		
	No	201 (97.1)		
Specific comorbidity presented	HIV	3 (1.4)		
	Hypertension	2 (1.0)		
	Diabetes mellitus and hypertension	1 (0.5)		
Treatment modality	Topical corticosteroids	174(84.1)		
	Systemic agent	22(10.6)		

	Topical corticosteroids and systemic agents	11(5.3)		

The majority of study populatition, 145 (70.0%) had plaque psoriasis followed by sebopsoriasis 24 (11.6%). In the present study, 158 (76.3%) patients were in the normal body mass index (BMI) range and only 6 (2.9%) patients had comorbid diseases among which human immunodeficiency virus (HIV) and hypertension accounted for 1.4% and 1.0% respectively.

Anti-psoriatic drug utilization practice

In our study, 185 (89.4%) of patients received topical corticosteroids for the treatment of psoriasis, and 12(6.5%) patients have prescribed two topical steroids with different potency. Methotrexate was found to be prescribed for 33(15.9%) patients. The average number of antipsoriatic drugs prescribed per patient was 1.8(SD=0.7). Regarding the management of specific types of psoriasis, plaque psoriasis was mostly managed by topical therapy alone, topical corticosteroids with or without salicylic acid/coal tar, 113 (77.9%) but only 21(14.5%) treated by methotrexate alone. All patients with palmoplantar, sebopsoriasis, scalp, and pustular psoriasis were treated only by topical corticosteroids with or without salicylic acid.

Table 3: Anti-psoriatic drugs utilization pattern among psoriasis patients attending ALERT Center, Addis Ababa, Ethiopia (N=207).

**Treatments**	**Types of Psoriasis, N (%)**						**Total N (%)**
	Plaque N=145	Inverse and nail N=1	Palmoplantar N=11	Sebopsoriasis N=26	Scalp N=22	Pustular N=2	
**Topical corticosteroids**							185(89.4)
Betamethasone dipropoinate	106(73.1)	0(0)	2(18.2)	22(84.6)	20(90.9)	2(100)	150(81.1)
Clobetasone	5(3.4)	0(0)	6(54.5)	0(0)	0(0)	0(0)	12(6.5)

propionate							
Mometasone furoate	11(7.6)	0(0)	2(18.2)	4(16.7)	0(0)	0(0)	17(9.2)
Clocortolone pivolate	8(5.5)	0(0)	3(27.3)	0(0)	2(9.1)	0(0)	12(6.5)
**Other topical agents**							
Salicylic acid	86(59.3)	0(0)	7(63.6)	16(66.7)	6(27.3)	0(0)	115(55.6)
Coal tar	8(5.5)	0(0)	0(0)	0(0)	0(0)	0(0)	8(3.9)
**Systemic agents (traditional)**							
Methotrexate	32(22.1)	1(100)	0(0)	0(0)	0(0)	0(0)	33(16.0)
**Mean ± SD**							
No. of drugs	1.8±0.7	1.0±0.0	2.0±0.7	2.5± 0.7	1.4±0.5	1.0±0.0	1.8±0.7
No. of preparation	1.2±0.4	1.0±0.0	1.2±0.4	1.7±0.5	1.1±0.3	1.0±0.0	1.2±0.4

Quality of Life Domains

According to DLQI, 104 (50.2%) patients experienced a small effect and only 4(2.0%) patients had an extremely large effect on the quality of life (Fig 1).

Figure 1: Proportion of overall effect of psoriatic on quality of life among psoriasis patients attending ALERT Hospital, Addis Ababa, Ethiopia (N=207).

Among DLQI domains, symptom and feeling was the highest affected domain, with a total value of 541, followed by treatment, with a total value of 287, followed by daily activities, with a total value of 232 and the least affected domain was work and school, with a total value of 54 (Table 4). The mean DLQI was 6.25 corresponding to a moderate effect and symptoms and feeling were the most affected domains of QoL.

Table 4: Quality of life domains of psoriatic patients attending Dermatologic Clinic of ALERT Hospital, Addis Ababa, Ethiopia (N=207).

**Domains**	**Question Number**	**Mean**	**Minimum value**	**Maximum value**	**Total value**
Symptoms and feelings	1	1.84	0	3	380
	2	0.78	0	3	161
Daily activities	3	0.22	0	3	45
	4	0.90	0	3	187
Leisure	5	0.23	0	3	47
	6	0.22	0	3	46
Work and School	7	0.25	0	3	51
		0.02	0	1	3
Personal relationships	8	0.31	0	3	64
	9	0.11	0	3	23
Treatment	10	1.39	0	3	287
**Overall DLQI**		6.25	0	29	1294

Factors associated with quality of life

Based on the results of univariate binary logistic regression analysis, variables such as gender, age group, educational status, occupation, family income, regular use of alcohol, BMI, age at initial diagnosis, and treatment modality were included in the multivariate binary logistic regression analysis, and accordingly, gender, educational status, family income, and treatment modality had a significant association with quality of life. The odds of having better QoL (DLQI≤5) for female patients was decreased by 83% [AOR=0.17 (95% CI: 0.06, 0.48)], (P=0.001).This study also found a statistically significant association between primary educational status and poor quality of life [AOR=0.14 (95% CI: 0.03, 0.64)] (P=0.011). The odds

of having better QoL (~~DLQI≤5~~) were more than four times higher among patients on systemic therapy as compared to those on topical only [AOR=4.26 (CI:1.18,15.35)] (P=0.027) (Table 5).

Table 5: Logistic regression analysis of factors associated with QoL among psoriasis patients attending Dermatologic Clinic of ALERT Hospital, Addis Ababa, Ethiopia (N=207).

**Variables**	**DLQI, n (%)**		**OR (95% CI)**		**P-value**
	**≤5**	**>5**	**Crude**	**Adjusted**	
**Sex**					
Male	35(41.18)	50(58.82)	1.00	1.00	
Female	82(67.21)	40(32.79)	0.34(0.19,0.61)	0.17(0.06,0.48)	0.001*
**Age group**					
16-35	66(62.26)	40(37.74)	1.00	1.00	
36-65	44(58.67)	31(41.33)	1.16(0.64,2.13)	0.92(0.33,2.60)	0.872
>65	7(26.92)	19(73.08)	4.48(1.73,11.60)	5.50(0.68,44.27)	0.109
**Educational status**					
Can't read and write	18(46.15)	21(53.85)	1.00	1.00	
Primary	38(82.61)	8(17.39)	0.18(0.07,0.49)	0.14(0.03,0.64)	0.011*
Secondary	22(50)	22(50)	0.86(0.36,2.03)	3.26(0.65,16.50)	0.152
Higher	39(50)	39(50)	0.86(0.40,1.85)	4.18(0.75,23.62)	0.102
**Occupation**					
Farmer	11(45.83)	13(54.17)	1.00	1.00	
Merchant	33(71.74)	13(28.26)	0.33(0.12,0.93)	0.87(0.16,4.56)	0.865
Employee	27(50.94)	26(49.06)	0.82(0.31,2.14)	1.28(0.23,7.07)	0.774
Unemployed	5(35.71)	9(64.29)	1.52(0.39,5.91)	1.89(0.22,16.00)	0.560
House wife	14(63.64)	8(36.36)	0.48(0.15,1.58)	4.80(0.78,29.60)	0.091
Student	21(72.41)	8(27.59)	0.32(0.10,1.01)	2.32(0.26,20.80)	0.450
Daily laborer	4(40)	6(60)	1.27(0.28,5.68)	5.48(0.56,53.95)	0.145
Others	2(22.22)	7(77.78)	2.96(0.51,17.30)	0.94(0.04,20.11)	0.970

**Monthly family income (ETB)**					
Very low ≤860)	10(31.25)	22(68.75)	1.00	1.00	
Low (861-1500)	32(76.19)	10(23.81)	0.14(0.05,0.40)	0.12(0.02,0.56)	0.007*
Average (1501-3000)	8(33.33)	16(67.67)	0.91(0.29,2.82)	0.24(0.04,1.43)	0.117
Above average (3001-5000)	19(59.38)	13(40.62)	0.31(0.11,0.87)	0.06(0.01,0.32)	0.001*
High (≥5001)	48(62.34)	29(37.66)	0.28(0.11,0.66)	0.03(0.01,0.22)	<0.001*
**Regular use of alcohol**					
No	115(57.79)	84(42.21)	1.00	1.00	
Yes	2(25)	6(75)	4.11(0.81,20.85)	8.05(0.55,117.65)	0.128
**BMI**					
Underweight	3(20)	12(80)	1.00	1.00	
Normal	93(58.86)	65(41.14)	0.18(0.05,0.64)	0.28(0.06,1.36)	0.114
Overweight	7(41.18)	10(58.82)	0.36(0.07,1.75)	0.86(0.10,7.31)	0.889
**Age at initial diagnosis (in years)**					
<40	90(62.5)	54(37.5)	1.00	1.00	
≥ 40	27(42.86)	36(57.14)	2.22(1.22,4.06)	1.82(0.47,7.04)	0.384
**Treatment modality**					
Topical	104(59.43)	71(40.57)	1.00	1.00	
Systemic	7(33.33)	14(67.67)	2.93(1.13,7.62)	4.26(1.18,15.35)	0.027*
Topical and systemic	6(54.54)	5(45.46)	1.22(0.36,4.15)	0.73(0.10,5.61)	0.765

*- statistically significant at P<0.05

Discussion

In this study, different medication regimens were used in the management of psoriasis. Topical corticosteroids were the most commonly prescribed medication, followed by methotrexate, in treating plaque psoriasis. Topical corticosteroids having anti-inflammatory, antiproliferative, immunosuppressive, and vasoconstrictive effects hence considered as a cornerstone of treatment

in those patients with the limited disease [19]. Similarly, methotrexate is folate synthesis inhibitor, used for the treatment of psoriasis due to its cytostatic and anti-inflammatory properties [3]. Likewise, different guidelines regarding the treatment of psoriasis vulgaris have shown that topical corticosteroids and methotrexate are one of the first lines for treating mild and moderate-severe psoriasis respectively [20-24]. Corticosteroids can even be used as an adjunct to systemic therapy or phototherapy for moderate-to-severe psoriasis [5,24]. Among patients with plaque psoriasis, extemporaneous preparation of salicylic acid with topical corticosteroids was prescribed for 86 (59.3%) patients. This could be due to the fact that salicylic acid, a keratolytic agent by itself, enhances steroid efficacy by increasing penetration [3,21]. Palmoplantar psoriasis, sebopsoriasis and scalp psoriasis were managed by topical corticosteroids alone or in combination with salicylic acid. This is in agreement with different clinical guidelines [22, 23].

Regarding the management of pustular psoriasis, the present study revealed that topical corticosteroids were the most commonly used treatment regimen. Contrastingly, clinical guideline done by National Psoriasis Foundation [22] did not state topical corticosteroids among both first and second-line therapy for treating such patients but rather recommended biologics. The probable reasons can be unavailability of biologics and lack of local standard treatment guidelines for the management of psoriasis in the studied hospital. Extemporaneous preparation containing coal tar with topical corticosteroids was prescribed for 8(3.9%) patients. Guideline on the management of psoriasis in South Africa showed that coal tar is recommended in patients with mild psoriasis who require an inexpensive alternative to corticosteroids [21]. Various guidelines also exposed that coal tar can be used for treating localized psoriasis, but it has poor tolerability by patients due to its irritation property, cosmetic issues like odor, and staining of clothes [5, 19,23]. So, patients prescribed for coal tar need higher attention.

In the present study, the mean DLQI was found to be 6.25, corresponding to a moderate effect of psoriasis on patients' QoL. The result was somewhat similar to a hospital-based cross-sectional study done in India [24], Malaysia [25], and Russia [26], which reported 8.10, 8.5, and 7.1 respectively. However, Egyptian study found a higher mean DLQL (12.2%), corresponding to a very large effect [27]. The first reason for this variation could be our study included all types of psoriasis, unlike the study done in Egypt which incorporated only psoriasis of chronic plaque-type and the second reason might be the difference in the severity of the disease among study participants as far as this study was carried out among outpatients only.

In the present study, only 13 (6.3%) patients replied that the disease has no effect on their quality of life which is similar to a report of Indian study, 6.1% (24). Among DLQI domains symptoms and feelings were the highly affected domain. The present result is in line with studies done in India [24,28], Taiwan [29], and Egypt [27]. This is due to the fact that the domain encompasses questions directed to identify the level of embarrassment and painfulness or itchiness owing to the disease which is used to even define the disease in the case of global reports on psoriasis [8].

The identification of risk factors for a marked reduction in quality of life owing to psoriasis is important as it helps to identify the most susceptible patient who requires close monitoring. The result of this study showed female gender was found to be independent predictors, which increases the chance of having poor quality of life. This was supported by a similar study done in India [30], the United States of America [31], Spain [32]. The probable reason could be the fact that females are more concerned about their body image and physical appearance than their male counterparts and they are more likely to feel troubled in public settings.

In this study, those patients with ~~primary education status~~, and having low, above average and high family monthly income were significantly associated with poor QoL which is contrary to a study done in India [33]. Such discrepancy might result from a difference in cultural settings, adjustment of confounding factors, DLQI cut-off point, and analysis model. Patients taking systemic therapy were significantly associated with less affected quality of life which might be owing to its more aggressive, comfortable, and less time-consuming nature as compared to topical therapy. This result is supported by findings from a cohort study in Spain [33].

Our study had some limitations. The temporal relationship between the dependent and independent variables does not be allowed due to cross-sectional nature of the study design. The other limitation is that the drugs considered during analysis were those prescribed at the time of recent follow-up; it didn't include drugs that were switched or withdrawn. This study also considered only pharmacologic interventions due to poor recording practice of the hospital.

Conclusion

Topical corticosteroids were the most commonly prescribed treatment modality, which accounted for 89.4%, for the treatment of psoriasis. Mean DLQI was 6.25, corresponding to a moderate effect on the quality of life, while symptoms and feelings were the most highly affected domain. Female gender, primary education level, and having low, above average, and high monthly family income were significantly associated with markedly reduced quality of life while receiving systemic therapy significantly associated with a better quality of life. Hence, future interventions targeting improving QoL and widening treatment options for psoriasis deemed to be necessary.

Acknowledgments

We acknowledge ALERT Center for allowing us to conduct this study. The authors also would like to acknowledge all participants, data collectors, staff working in the dermatologic clinic of ALERT Center for their time, and voluntary facilitation for the data collection process.

Author Contributions

Conceptualization: Seifu Megersa Kumsa, Tamrat Assefa Tadesse, Minyahil Alebachew Woldu

Data curation: Seifu Megersa Kumsa

Formal analysis: Seifu Megersa Kumsa

Investigation Seifu Megersa Kumsa, Tamrat Assefa Tadesse, Minyahil Alebachew Woldu

Supervision: Tamrat Assefa Tadesse, Minyahil Alebachew Woldu

Writing – original draft: Seifu Megersa Kumsa

Writing – review & editing: Tamrat Assefa Tadesse, Minyahil Alebachew Woldu

Abbreviations

AOR: Adjusted Odds Ratio

ALERT- All Africa Leprosy, Tuberculosis and Rehabilitation Training

DLQI- Dermatology life quality index

QoL- Quality of life

SPSS: Statistical Package for Social Sciences (SPSS)

References

1. Belinchon I, Rivera R, Blanch C, Comellas M, Lizan L. Adherence , satisfaction and preferences for treatment in patients with psoriasis in the European Union : a systematic review of the literature. Patient Prefer Adherence. 2016; 10: 2357-67..
2. Boehncke W-H, Schon MP. Psoriasis. Seminar. 2015;983–94.
3. Kim WB, Jerome D, Yeung J. Diagnosis and management of psoriasis. Clin Rev. 2017;63:278–85.
4. Huerto C, Rivero E, Rodriguez LAG. Incidence and Risk Factors for Psoriasis in the General Population. Arch Dermatol. 2007;143:1559–65.
5. Lebwohi M. Psoriasis. Ann Intern Med. 2018; 168: ITC49-64.
6. Voorhees AS Van, Feldman SR, Lebwohl MG, Mandelin A, Ritchlin C. The psoriasis and psoriatic arthritis treatment algorithms and management options. National Psoriasis Foundation. 5th edition.
7. Schadler DC , Ortel B, Mehlis LS. Disease-a-Month Biologics for the primary care physician : Review and treatment of psoriasis. Disease-a-Month. 2018; 0:1–40.
8. World Health Organization. Global report on psoriasis. 2016; Available from: https://apps.who.int/iris/handle/10665/204417. Accessed on May 25 2019.
9. García-sánchez L, Montiel-jarquín ÁJ, Vázquez-cruz E, May-salazar A. Quality of life in patients with psoriasis.Gac Med Mex. 2017; 153:185–9.
10. Gottlieb AB. Therapeutic options in the treatment of psoriasis and atopic dermatitis. J Am Acedamy Dermatol. 2005;53:S3–16.
11. Fatani MI, Habibullah TH, Alfif AA, Ibrahim II, Althebyani B. Impact of Psoriasis on Quality of Life at Hera General Hospital in Makkah , Saudi Arabia. Clin Med Diagnostics. 2016;6: 7-12.
12. Palijan T, Koi E, Ru K, Dervinja F. The Impact of Psoriasis on the Quality of Life and Psychological Characteristics of Persons Suffering from Psoriasis. Coll Antropol. 2011;35:81–5.
13. Colombo D, Perego R. Quality of Life in Psoriasis. Intechopen. 2013;142–9.
14. Mendoza-sassi RA. Impact on the quality of life of dermatological patients in Southern Brazil. An Bras Dermattol. 2011;86:1113-21.
15. Bhosle MJ, Kulkarni A, Feldman SR, Balkrishnan R. Quality of life in patients with

psoriasis. Health Qual Life Outcomes. 2006;7:1–7.

16. Abdulghani M, Sheik a al, Alkhawajah M, Ammoury A, Behrens F, Benchikhi H, et al. Management of Psoriasis in Africa and the Middle East : a Review of Current Opinion , Practice and Opportunities for Improvement. J Int Med Res. 2011;1573–88.
17. Kragballe UMK, Spuls KRP, Nast CEMGA, Antoniou JFC. Definition of treatment goals for moderate to severe psoriasis : a European consensus. Arch Dermatol Res. 2011;1–10.
18. Cardiff University. Dermatology life quality index [Internet]. 1994. Available from: https://www.cardiff.ac.uk/medicine/resources/quality-of-life-questionnaires/dermatology-life-quality-index
19. Menter A, Korman NJ, Elmets CA, Feldman SR, Gelfand JM, Gordon KB, et al. Guidelines of care for the management of psoriasis and psoriatic arthritis. J Am Dermatology. 2009;60:643–59.
20. Alexander Nast, Boehncke W, Mrowietz U, Ockenfels H, Philipp S, Reich K, et al. European S3 guidelines on treatment of psoriasis vulgaris. J Ger Soc Dermatol. 2012; 51-95
21. Raboobee N, Group W, Aboobaker J, Jordaan HF, Sinclair W, Smith JM, et al. Guideline on the management of psoriasis in South Africa. S Afr Med J. 2010;100:257-82.
22. National Psoriasis Foundation. The psoriasis and psoriatic arthritis. 2016;5th edition.
23. Papp K, Gulliver W,Lynde C, Poulin Y, Ashkenas J. Canadian Guidelines for the Management of Plaque Psoriasis. J Cutan Med Surg. 2011; 15: 210-9.
24. Vettuparambil A, Neelakandhan A. Psoriasis can markedly impair the quality of life of patients irrespective of severity : Results of a hospital-based cross-sectional study. 2016;7: 111-4.
25. Saaya NN, Khan I, Affandi AM. Epidemiology and Clinical Features of Adult Patients with Psoriasis in Malaysia : 10-Year Review from the Malaysian Psoriasis Registry (2007 – 2016). Hindawi. 2018; 2018: 1-8.
26. Kubanov AA, Bakulev AL, Fitileva T V., Novoderezhkina E, Gilloteau I, Tian H, et al. isease Burden and Treatment Patterns of Psoriasis in Russia : A Real-World Patient and Dermatologist Survey. Dermatol Ther (Heidelb). 2018; 8:581–92.
27. Eid AA, Elweshahi HM. Quality of life of Egyptian patients with psoriasis : a hospital- based cross-sectional survey. Egypt J Dermatology Venereol. 2016;36:11–7.
28. Barot PA,Brahmbhatt NA, , Malhotra Quality of life in patients with psoriasis at a tertiary

care teaching hospital – a cross sectional study. Natl J Med Res. 2015;5:93–7.
29. Lin T, See L, Shen Y, Liang C, Chang H, Lin Y. Quality of Life in Patients with Psoriasis in Northern Taiwan. Chang Gung Med J. 2010;186–96.
30. Nagrani P, Roy S, Jindal R. Quality of life in psoriasis : a clinical study. Int J Res Dermatol. 2019;5:1–6.
31. Rolstad T, Gelfand JM, Feldman SR, S.Stern R, Thomas J, Margolis DJ. Determinants of quality of life in patients with psoriasis: A study from the US population. Am Acad Dermatology. 2004;704–8.
32. Pita-Fernandez S, Torres RMF, Fonseca E. Quality of life and related factors in a cohort of plaque-type psoriasis patients in La Coruna. Int J Dermatol. 2014;53: 507–11.
33. Nayak, Girisha, Noronha. Correlation between Disease Severity, Family Income, and Quality of Life in Psoriasis: A Study from South India. Indian Dermatol Online J. 2018;9:165-9.

 Click here to access/download;Figure;Fig 1.docx

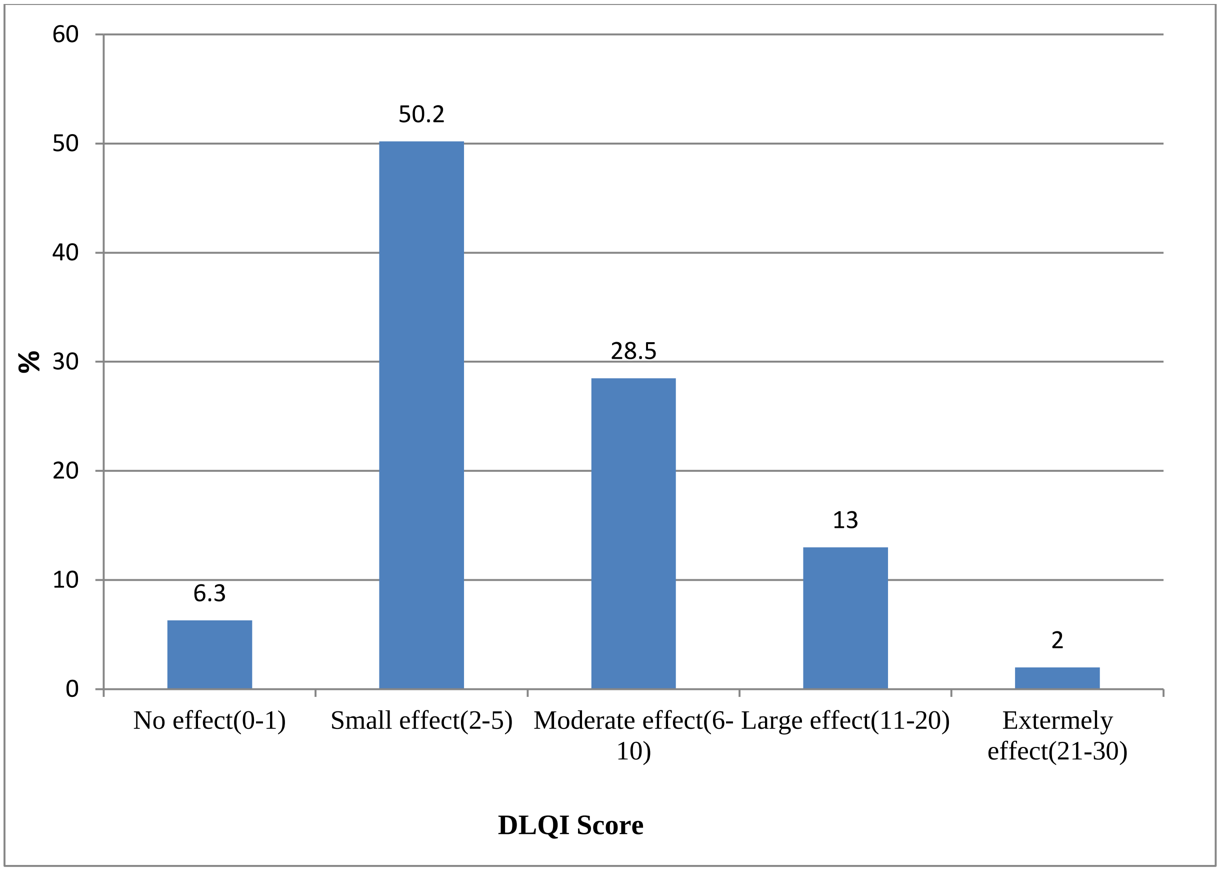

Supplement: Attachment — Submitted filename: PONE-D-20-33787 with remarks.pdf [file pone.0260243.s005.pdf]
