# Supplementary material for: Management practice, quality of life and associated factors in psoriasis patients attending a dermatological center in Ethiopia
Source: PLoS One. 2021 Nov 19;16(11):e0260243. doi: 10.1371/journal.pone.0260243 (PMC8604307; doi:10.1371/journal.pone.0260243)
Supplement: Attachment — Submitted filename: PONE-D-20-33787_review.docx [file pone.0260243.s004.docx]

In this manuscript for a research article, authors investigated the clinical care, quality of life and various associated factors of psoriatic patients managed at an outpatient clinic located in Addis Ababa, Ethiopia. As previous data published on the management of psoriasis in East Africa is very limited, the topic and findings of this paper is interesting. However, there are some major issues to be clarified by the authors.

Major remarks:

1. The exact inclusion and exclusion criteria of this study should be described instead of just reporting general eligibility criteria.
2. It is very surprising data that only 2.9% of the patients had comorbid diseases. Were the general medical records of the patients also reviewed? These data should be also discussed.
3. It should be described in the Introduction, which topicals, drugs and biologics are available for the treatment of psoriasis in Ethiopia, as treatment choices also highly depends on the availability of medications.
4. In the Discussion, it its not necessary to describe how corticosteroids and methotrexate work but would be important to compare the used treatment modalities to those of other regions and countries. Also, the benefits and limitations of inexpensive treatment options such as coal tar ointments should be discussed.

Minor remarks:

1. In the abstract, it is not necessary to include technical details, such as the name of the exact statistical software used for data analysis.
2. In the Introduction, epidemiologic data of psoriasis is only mentioned for Asia and Europe. It would be more appropriate to describe epidemiologic data from Africa. Also, previous papers describing the care for psoriatic patients in East Africa should be cited here.
3. In the Study setting section, it is confusing that only some general facts on the department, such as when it was established and in which medical specialties it is a leading center is reported. The prospective nature of the study should be described here.
4. The price of each treatment option should be also discussed in addition to their benefit in QOL, especially as the income of the patients was also reported.
5. Further conclusions should be added based on the observations of this study instead of repetition of the results.
